# End-Completely-Regular and End-Inverse Lexicographic Products of Graphs

**DOI:** 10.1155/2014/432073

**Published:** 2014-04-17

**Authors:** Hailong Hou, Rui Gu

**Affiliations:** School of Mathematics and Statistics, Henan University of Science and Technology, Luoyang, Henan 471003, China

## Abstract

A graph *X* is said to be End-completely-regular (resp., End-inverse) if its endomorphism monoid End(*X*) is completely regular (resp., inverse). In this paper, we will show that if *X*[*Y*] is End-completely-regular (resp., End-inverse), then both *X* and *Y* are End-completely-regular (resp., End-inverse). We give several approaches to construct new End-completely-regular graphs by means of the lexicographic products of two graphs with certain conditions. In particular, we determine the End-completely-regular and End-inverse lexicographic products of bipartite graphs.

## 1. Introduction and Preliminary Concepts

Endomorphism monoids of graphs are generalizations of automorphism groups of graphs. In recent years, much attention has been paid to endomorphism monoids of graphs and many interesting results concerning graphs and their endomorphism monoids have been obtained. The aim of this research is to develop further relationship between graph theory and algebraic theory of semigroups and to apply the theory of semigroups to graph theory. The bipartite graph is a class of famous graphs. Their endomorphism monoids are studied by several authors. In [1], the connected bipartite graphs whose endomorphism monoids are regular were explicitly found. In [2], Fan gave a characterization of connected bipartite graphs with an orthodox monoid. The joins of bipartite graphs with regular endomorphism monoids were characterized in [3]. The joins of bipartite graphs with completely regular endomorphism monoids were characterized in [4]. The endomorphism monoids and endomorphism regularity of graphs were considered by several authors (see [5–8]). In this paper, we will characterize the End-completely-regular and End-inverse lexicographic products of two graphs. We give several approaches to construct new End-completely-regular graphs by means of the lexicographic products of two graphs with certain conditions. In particular, we will determine the End-completely-regular and End-inverse lexicographic products of bipartite graphs.

The graphs *X* considered in this paper are undirected finite simple graphs. The vertex set of *X* is denoted by *V*(*X*) and the edge set of *X* is denoted by *E*(*X*). If two vertices *x*_1_ and *x*_2_ are adjacent in *X*, the edge connecting *x*_1_ and *x*_2_ is denoted by {*x*_1_, *x*_2_} and we write {*x*_1_, *x*_2_} ∈ *E*(*X*). A subgraph *H* is called an* induced subgraph* of *X* if for any *a*, *b* ∈ *V*(*H*), {*a*, *b*} ∈ *E*(*H*) if and only if {*a*, *b*} ∈ *E*(*X*). A graph *X* is called* bipartite* if *X* has no odd cycle. It is known that if a graph *X* is a bipartite graph, then its vertex set can be partitioned into two disjoint nonempty subsets such that no edge joins two vertices in the same set.

Let *X* and *Y* be two graphs. The* join* of *X* and *Y*, denoted by *X* + *Y*, is a graph such that *V*(*X* + *Y*) = *V*(*X*) ∪ *V*(*Y*) and *E*(*X* + *Y*) = *E*(*X*) ∪ *E*(*Y*)∪{{*x*_1_, *x*_2_} | *x*_1_ ∈ *V*(*X*), *x*_2_ ∈ *V*(*Y*)}. The* lexicographic product* of *X* and *Y*, denoted by *X*[*Y*], is a graph with vertex set *V*(*X*[*Y*]) = *V*(*X*) × *V*(*Y*), and with edge set *E*(*X*[*Y*]) = {{(*x*, *y*), (*x*_1_, *y*_1_)}∣{*x*, *x*_1_} ∈ *E*(*X*), or *x* = *x*_1_ and {*y*, *y*_1_} ∈ *E*(*Y*)}. Denote *Y*_*x*_ = {(*x*, *y*) | *y* ∈ *V*(*Y*)} for any *x* ∈ *V*(*X*).

Let *X* and *Y* be graphs. A mapping *f* from *V*(*X*) to *V*(*Y*) is called a* homomorphism* (from *X* to *Y*) if {*x*_1_, *x*_2_} ∈ *E*(*X*) implies that {*f*(*x*_1_), *f*(*x*_2_)} ∈ *E*(*Y*). A homomorphism *f* is called an* isomorphism* if *f* is bijective and *f* ^−1^ is a homomorphism. A homomorphism (resp., isomorphism) *f* from *X* to itself is called an* endomorphism* (resp.,* automorphism*) of *X* (see [9]). The sets of all endomorphisms and automorphisms of *X* are denoted by End(*X*) and Aut(*X*), respectively. A graph *X* is said to be* unretractive* if End(*X*) = Aut(*X*). For any *f* ∈ End(*X*), it is easy to see that *f* ∈ Aut(*X*) if and only if *f* is injective.

A* retraction* of a graph *X* is a homomorphism *f* from *X* to a subgraph *Y* of *X* such that the restriction *f*|_*Y*_ of *f* to *V*(*Y*) is the identity mapping on *V*(*Y*). In this case, *Y* is called a* retract* of *X*. It is known that the idempotents of End(*X*) are retractions of *X*. Denote by Idpt(*X*) the set of all idempotents of End(*X*). Let *f* be an endomorphism of a graph *X*. A subgraph of *X* is called the* endomorphic image* of *X* under *f*, denoted by *I*_*f*_, if *V*(*I*_*f*_) = *f*(*V*(*X*)) and {*f*(*a*), *f*(*b*)} ∈ *E*(*I*_*f*_) if and only if there exist *c* ∈ *f* ^−1^(*f*(*a*)) and *d* ∈ *f* ^−1^(*f*(*b*)) such that {*c*, *d*} ∈ *E*(*X*). By *ρ*_*f*_ we denote the equivalence relation on *V*(*X*) induced by *f*; that is, for *a*, *b* ∈ *V*(*X*), (*a*, *b*) ∈ *ρ*_*f*_ if and only if *f*(*a*) = *f*(*b*). Denote by [*a*]_*ρ*_*f*__ the equivalence class containing *a* ∈ *V*(*X*) with respect to *ρ*_*f*_.

An element *a* of a semigroup *S* is called* regular* if there exists *x* ∈ *S* such that *axa* = *a*. An element *a* of a semigroup *S* is called* completely regular* if *a* = *axa* and *xa* = *ax* hold for some *x* ∈ *S*. A semigroup *S* is called* regular* (resp.,* completely regular*) if all its elements are regular (resp., completely regular). An* inverse semigroup* is a regular semigroup in which the idempotents commute. A graph *X* is said to be End-regular (resp., End-completely-regular, End-inverse) if its endomorphism monoid End(*X*) is regular (resp., completely regular, inverse). Clearly, End-completely-regular graphs as well as End-inverse graphs are End-regular.

For undefined notation and terminology in this paper, the reader is referred to [9–14]. We list some known results which will be used in the sequel.

**Lemma 1 (see [2])**

If *X*[*Y*] is End-regular, then both *X* and *Y* are End-regular.

**Lemma 2 (see [15])**

Let *G* be a graph and let *f* ∈ *End*(*G*). Then *f* is completely regular if and only if *f*|_*I*_*f*__ ∈ *Aut*(*I*_*f*_).

**Lemma 3 (see [15])**

Let *X* be a bipartite graph. Then *X* is End-completely-regular if and only if *X* is one of *K*_1_, *K*_2_, *P*_2_, 2*K*_1_, 2*K*_2_, and *K*_1_ ⋃ *K*_2_.

**Lemma 4 (see [4])**

Let *X* and *Y* be two bipartite graphs. Then *X* + *Y* is End-completely-regular if and only if one of them is End-completely-regular and the other is *K*_1_ or *K*_2_.

**Lemma 5 (see [16])**

Let *G* be a graph and *f* ∈ *End*(*G*). Then *f* is completely regular if and only if there exists *g* ∈ *Idpt*(*G*) such that *ρ*_*g*_ = *ρ*_*f*_ and *I*_*g*_ = *I*_*f*_.

**Lemma 6 (see [4])**

Let *X* be a bipartite graph. Then *X* is End-inverse if and only if *X* = *K*_1_ or *X* = *K*_2_.

**Lemma 7 (see [17])**

Let *X* and *Y* be two graphs. Then *End*(*X*[*Y*]) = *End*(*X*)[*End*(*Y*)] if and only if for any *f* ∈ *End*(*X*[*Y*]) and *x* ∈ *V*(*X*), there exists *x*′ ∈ *V*(*X*) such that *f*(*Y*_*x*_)⊆*Y*_*x*′_.

## 2. Main Results

In this section, we will characterize the End-completely-regular and End-inverse lexicographic products of two graphs. We first show that if *X*[*Y*] is End-completely-regular, then both *X* and *Y* are End-completely-regular.

**Theorem 8**

Let *X* and *Y* be two graphs. If *X*[*Y*] is End-completely-regular, then both *X* and *Y* are End-completely-regular.

**Proof**

By Lemma 2, to show that *X* is End-completely-regular, it is only necessary to verify that *f*|_*I*_*f*__ is an automorphism of *I*_*f*_ for each *f* ∈ End(*X*). Define a mapping *F* from *V*(*X*[*Y*]) to itself by

(1) $$\begin{array}{c} F\left(\left(x,y\right)\right)=\left(f\left(x\right),y\right)\;\forall \left(x,y\right)\in V\left(X\left[Y\right]\right). \end{array}$$

Then *F* ∈ End(*X*[*Y*]). Since *X*[*Y*] is End-completely-regular, by Lemma 2, *F*|_*I*_*F*__ is an automorphism of *I*_*F*_. It is easy to see that *I*_*F*_ = *I*_*f*_[*Y*]. For any distinct *x*_1_, *x*_2_ ∈ *V*(*I*_*f*_) and *y* ∈ *V*(*Y*), *F*((*x*_1_, *y*)) = (*f*(*x*_1_), *y*) and *F*((*x*_2_, *y*)) = (*f*(*x*_2_), *y*) hold. Since *F*|_*I*_*F*__ is an automorphism of *I*_*F*_, (*f*(*x*_1_), *y*)≠(*f*(*x*_2_), *y*). Hence *f*(*x*_1_) ≠ *f*(*x*_2_) and so *f*|_*I*_*f*__ is an automorphism of *I*_*f*_.

Let *g* ∈ End(*Y*). Define a mapping *G* from *V*(*X*[*Y*]) to itself by

(2) $$\begin{array}{c} G\left(\left(x,y\right)\right)=\left(x,g\left(y\right)\right)\;\forall \left(x,y\right)\in V\left(X\left[Y\right]\right). \end{array}$$

Then *G* ∈ End(*X*[*Y*]). Since *X*[*Y*] is End-completely-regular, by Lemma 2, *G*|_*I*_*G*__ is an automorphism of *I*_*G*_. It is easy to see that *I*_*G*_ = *X*[*I*_*g*_]. For any *x* ∈ *V*(*X*) and *y*_1_, *y*_2_ ∈ *V*(*I*_*g*_), *G*((*x*, *y*_1_)) = (*x*, *g*(*y*_1_)) and *G*((*x*, *y*_2_)) = (*x*, *g*(*y*_2_)). Since *G*|_*I*_*G*__ is an automorphism of *I*_*G*_, (*x*, *g*(*y*_1_))≠(*x*, *g*(*y*_2_)), we get that *g*(*y*_1_) ≠ *g*(*y*_2_) and so *g*|_*I*_*g*__ is an automorphism of *I*_*g*_, as required.

The following example shows that *X* and *Y* being End-completely-regular does not yield that *X*[*Y*] is End-completely-regular.

**Example 9**

Let *X* and *Y* be two graphs with *V*(*X*) = {*x*_1_, *x*_2_}, *V*(*Y*) = {*y*_1_, *y*_2_}, *E*(*X*) = {{*x*_1_, *x*_2_}}, and *E*(*Y*) = *ϕ*. By Lemma 3, *X* and *Y* are End-completely-regular. It is easy to see that *X*[*Y*]≅*C*_4_. Also by Lemma 3, this is not End-completely-regular.

In the following, we give some sufficient conditions for *X*[*Y*] to be End-completely-regular. To this aim, we need the following result due to Fan [17].

**Lemma 10 (see [17])**

Let *X* and *Y* be two *K*_3_-free connected graphs. If *girth*(*X*) or *girth*(*Y*) is odd, then *End*(*X*[*Y*]) = *End*(*X*)[*End*(*Y*)], where *End*(*X*)[*End*(*Y*)] is the wreath product of the monoids *End*(*X*) and *End*(*Y*).

Let *X* and *Y* be two *K*_3_-free connected graphs such that girth(*X*) or girth(*Y*) is odd. In [2], Fan proved that if both of *X* and *Y* are End-regular and one of them is unretractive, then *X*[*Y*] is End-regular. Here we prove that if *X* is an End-completely-regular graph and *Y* is an unretractive graph, then *X*[*Y*] is End-completely-regular.

**Theorem 11**

Let *X* and *Y* be two *K*_3_-free connected graphs with *girth*(*X*) or *girth*(*Y*) being odd, and assume that

1. *X*  is End-completely-regular,
2. *Y* is unretractive.

Then *X*[*Y*] is End-completely-regular.

**Proof**

Let *X* and *Y* be two graphs satisfying the assumptions. To show that *X*[*Y*] is End-completely-regular, we prove that for any *F* ∈ End(*X*[*Y*]), there exists an idempotent endomorphism *G* ∈ End(*X*[*Y*]) such that *ρ*_*F*_ = *ρ*_*G*_ and *I*_*F*_ = *I*_*G*_.

Let *F* ∈ End(*X*)[End(*Y*)]. Since End(*X*[*Y*]) = End(*X*)[End(*Y*)], *F* = (*s*, *f*) for some *s* ∈ End(*X*) and *f* ∈ End(*Y*)^*V*(*X*)^. Thus, for any *u* ∈ *V*(*X*), there exists *f*_*u*_ = *f*(*u*) ∈ End(*Y*). Let *X* and *Y* be *K*_3_-free connected graphs with girth(*X*) or girth(*Y*) being odd. By Lemma 7, for any *u* ∈ *V*(*X*), *F*(*Y*_*u*_)⊆*Y*_*v*_ for some *v* ∈ *V*(*X*). Note that *Y* is unretractive. Then *F*(*Y*_*u*_) = *Y*_*v*_. Since *X* is End-completely-regular and *s* ∈ End(*X*), by Lemma 5, there exists *t* ∈ Idpt(*X*) such that *ρ*_*t*_ = *ρ*_*s*_ and *I*_*t*_ = *I*_*s*_. Clearly, *I*_*s*_ is an induced subgraph of *X*. Hence *I*_*F*_ = *I*_*s*_[*Y*] is an induced subgraph of *X*[*Y*].

Since *X* is End-completely-regular, *s*|_*I*_*s*__ is an automorphism of *I*_*s*_. Thus for any *u* ∈ *I*_*s*_, there exists only one vertex *u*_1_ ∈ *I*_*s*_ such that *s*(*u*_1_) = *u*. Then *F*(*Y*_*u*_1__) = *Y*_*u*_. Now for any (*u*, *v*) ∈ *I*_*F*_, there exists only one vertex (*u*_1_, *v*_1_) ∈ *I*_*F*_ such that *F*((*u*_1_, *v*_1_)) = (*u*, *v*). Define a mapping *G* from *V*(*X*[*Y*]) to itself in the following way. If (*x*, *y*) ∈ *V*(*I*_*F*_), then *G*((*x*, *y*)) = (*x*, *y*); if (*x*, *y*) ∉ *V*(*I*_*F*_), then *F*((*x*, *y*)) = (*u*, *v*) for some (*u*, *v*) ∈ *V*(*I*_*F*_). Now let *G*((*x*, *y*)) = (*u*_1_, *v*_1_), where (*u*_1_, *v*_1_) is the only vertex in *V*(*I*_*F*_) such that *F*((*u*_1_, *v*_1_)) = (*u*, *v*). Then it is easy to see that *G* is well-defined. Let (*x*, *y*) ∈ *V*(*X*[*Y*]). If (*x*, *y*) ∈ *V*(*I*_*F*_), then *x* ∈ *I*_*s*_. Thus *t*(*x*) = *x*. Hence *G*((*x*, *y*)) = (*x*, *y*) ∈ *Y*_*t*(*x*)_. If (*x*, *y*) ∉ *V*(*I*_*F*_), then *t*(*x*) = *u*_1_. Hence *G*((*x*, *y*)) = (*u*_1_, *v*_1_) ∈ *Y*_*t*(*x*)_. Therefore, *G*((*x*, *y*)) ∈ *Y*_*t*(*x*)_ for any (*x*, *y*) ∈ *V*(*X*[*Y*]).

Let (*x*_1_, *y*_1_), (*x*_2_, *y*_2_) ∈ *V*(*X*[*Y*]) be such that {(*x*_1_, *y*_1_), (*x*_2_, *y*_2_)} ∈ *E*(*X*[*Y*]). If (*x*_1_, *y*_1_), (*x*_2_, *y*_2_) ∈ *V*(*I*_*F*_), then {*G*((*x*_1_, *y*_1_)), *G*((*x*_2_, *y*_2_))} = {(*x*_1_, *y*_1_), (*x*_2_, *y*_2_)} ∈ *E*(*X*[*Y*]). If (*x*_1_, *y*_1_) ∈ *V*(*I*_*F*_) and (*x*_2_, *y*_2_) ∉ *V*(*I*_*F*_), then *x*_1_ ≠ *x*_2_ and {*x*_1_, *x*_2_} ∈ *E*(*X*). Thus *G*((*x*_1_, *y*_1_)) ∈ *Y*_*t*(*x*_1_)_ and *G*((*x*_2_, *y*_2_)) ∈ *Y*_*t*(*x*_2_)_. Since *t* ∈ Idpt(*X*) and {*x*_1_, *x*_2_} ∈ *E*(*X*), {*t*(*x*_1_), *t*(*x*_2_)} ∈ *E*(*X*). Hence {*G*((*x*_1_, *y*_1_)), *G*((*x*_2_, *y*_2_))} ∈ *E*(*X*[*Y*]). If (*x*_1_, *y*_1_) ∉ *V*(*I*_*F*_) and (*x*_2_, *y*_2_) ∉ *V*(*I*_*F*_), there are two cases.

*Case 1.* Assume that {*x*_1_, *x*_2_} ∈ *E*(*X*). Then *G*((*x*_1_, *y*_1_)) ∈ *Y*_*t*(*x*_1_)_ and *G*((*x*_2_, *y*_2_)) ∈ *Y*_*t*(*x*_2_)_. Since *t* ∈ Idpt(*X*) and {*x*_1_, *x*_2_} ∈ *E*(*X*), {*t*(*x*_1_), *t*(*x*_2_)} ∈ *E*(*X*). Hence we have {*G*((*x*_1_, *y*_1_)), *G*((*x*_2_, *y*_2_))} ∈ *E*(*X*[*Y*]).

*Case 2.* Assume that *x*_1_ = *x*_2_ and {*y*_1_, *y*_2_} ∈ *E*(*Y*). Then we have (*x*_1_, *y*_1_), (*x*_2_, *y*_2_) ∈ *Y*_*x*_1__ and *G*((*x*_1_, *y*_1_)), *G*((*x*_2_, *y*_2_)) ∈ *Y*_*t*(*x*_1_)_. Since *G*|_*Y*_*x*_1___ is an isomorphism from *Y*_*x*_1__ to *Y*_*t*(*x*_1_)_, {*G*((*x*_1_, *y*_1_)), *G*((*x*_2_, *y*_2_))} ∈ *E*(*X*[*Y*]). Therefore, *G* ∈ End(*X*[*Y*]).

If (*x*, *y*) ∈ *V*(*I*_*F*_), then *G* ^2^((*x*, *y*)) = *G*((*x*, *y*)) = (*x*, *y*). If (*x*, *y*) ∉ *V*(*I*_*F*_), then *G*((*x*, *y*)) ∈ *V*(*I*_*F*_). Thus *G* ^2^((*x*, *y*)) = *G*(*G*((*x*, *y*))) = *G*((*x*, *y*)). Hence *G* ∈ Idpt(*X*[*Y*]). Clearly, *I*_*G*_ = *I*_*F*_.

Suppose [(*x*_1_, *y*_1_)]_*ρ*_*F*__ = {(*x*_1_, *y*_1_), (*x*_2_, *y*_2_),…, (*x*_*k*_, *y*_*k*_)} for some (*x*_1_, *y*_1_), (*x*_2_, *y*_2_),…, (*x*_*k*_, *y*_*k*_) ∈ *V*(*X*[*Y*]). In fact, it is easy to prove that *ρ*_*F*_⊆*ρ*_*G*_⊆*ρ*_*F*_. Let (*x*_1_, *y*_1_)*ρ*_*F*_(*x*_2_, *y*_2_). Then, by the definition of *G*, we have *G*((*x*_1_, *y*_1_)) = *G*((*x*_2_, *y*_2_)) = (*u*_1_, *v*_1_) for some (*u*_1_, *v*_1_) ∈ *V*(*I*_*F*_) with *F*((*x*_1_, *y*_1_)) = *F*(*u*_1_, *v*_1_) = *F*((*x*_2_, *y*_2_)). So (*x*_1_, *y*_1_)*ρ*_*G*_(*x*_2_, *y*_2_) and thus *ρ*_*F*_⊆*ρ*_*G*_⊆*ρ*_*F*_. Hence *ρ*_*F*_ = *ρ*_*G*_.

Next we start to seek the conditions for bipartite graphs *X* and *Y* under which *X*[*Y*] is End-completely-regular.

**Lemma 12**

Let *X* be a graph and *R* be a retract of *X*. If *R*[*Y*] is not End-completely-regular, then *X*[*Y*] is not End-completely-regular.

**Proof**

Let *R* be a retract of *X*. Then there exists *f* ∈ Idpt(*X*) such that *I*_*f*_ = *R*. Let *g* ∈ End(*R*[*Y*]). Since *R*[*Y*] is not End-completely-regular, there exists *g* ∈ End(*R*[*Y*]) such that *g* is not completely regular. By Lemma 2, *g*|_*I*_*g*__ is not an automorphism of *I*_*g*_. Thus there exist *x*_1_, *x*_2_ ∈ *I*_*g*_ with *x*_1_ ≠ *x*_2_ such that *g*(*x*_1_) = *g*(*x*_2_). Define a mapping *F* from *V*(*X*[*Y*]) to itself by

(3) $$\begin{array}{c} F\left(\left(x,y\right)\right)=\left(f\left(x\right),y\right)\;\forall \left(x,y\right)\in V\left(X\left[Y\right]\right). \end{array}$$

Then *F* ∈ End(*X*[*Y*]) and *I*_*F*_ = *R*[*Y*]. Now it is easy to see that *gF* ∈ End(*X*[*Y*]) and *I*_*gF*_ = *I*_*g*_. It follows from (*gF*)(*x*_1_) = (*gF*)(*x*_2_) that (*gF*)|_*I*_*gF*__ is not an automorphism of *I*_*gF*_. Hence *X*[*Y*] is not End-completely-regular.

**Lemma 13**

Let *X* and *Y* be two graphs. If at least one of *X* and *Y* is not End-completely-regular, then *X* ∪ *Y* is not End-completely-regular (where *X* ∪ *Y* is the disjoint union of *X* and *Y*).

**Proof**

Without loss of generality, we may suppose that *X* is not End-completely-regular. By Lemma 2, there exists *f* ∈ End(*X*) such that *f*|_*I*_*f*__ is not an automorphism of *I*_*f*_. Define a mapping *F* from *V*(*X* ∪ *Y*) to itself by

(4) $$\begin{array}{c} F\left(x\right)=\{\begin{array}{cc} f\left(x\right), & x\in V\left(X\right),\\ x, & x\in V\left(Y\right). \end{array} \end{array}$$

Then *F* ∈ End(*X* ∪ *Y*). Now it is easy to see that *I*_*F*_ = *I*_*f*_ ∪ *Y* and *F*(*x*) = *f*(*x*) for any *x* ∈ *V*(*X*). Since *f*|_*I*_*f*__ is not an automorphism of *I*_*f*_, *F*|_*I*_*F*__ is not an automorphism of *I*_*F*_. Hence *X* ∪ *Y* is not End-completely-regular.

**Theorem 14**

Let *X* and *Y* be two bipartite graphs. Then *X*[*Y*] is End-completely-regular if and only if

1. *X* = *K*_1_ and *Y* is End-completely-regular or
2. *X* is End-completely-regular and *Y* = *K*_1_ or *K*_2_.

**Proof**

**Figure 1:**
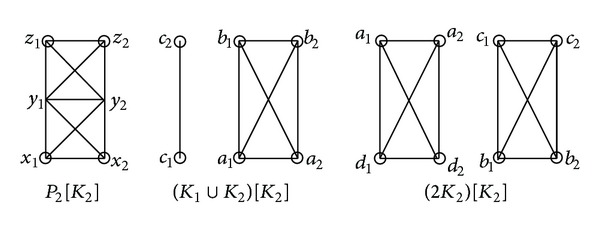
Graphs *P*_2_[*K*_2_], (*K*_1_ ∪ *K*_2_)[*K*_2_], and (2*K*_2_)[*K*_2_].

*Sufficiency.* Since *K*_1_[*Y*] = *Y* and *X*[*K*_1_] = *X*, we have immediately that *K*_1_[*Y*](*X*[*K*_1_]) is End-completely-regular if and only if *Y*(*X*) is End-completely-regular. If *X* = *Y* = *K*_2_, then *X*[*Y*] = *K*_4_. Thus End(*X*[*Y*]) is a group. Since any group is a completely regular semigroup, *X*[*Y*] is End-completely-regular. If *X* = 2*K*_1_ and *Y* = *K*_2_, then *X*[*Y*] = 2*K*_2_. By Lemma 3, it is End-completely-regular. In the following, we show that *X*[*Y*] is End-completely-regular for the following cases (see Figure 1).

*Case 1.* *X* = *P*_2_ and *Y* = *K*_2_. Let *f* ∈ End(*X*[*Y*]). If [*x*_1_]_*ρ*_*f*__ = {*x*_1_}, then [*x*_2_]_*ρ*_*f*__ = {*x*_2_}. Otherwise, *f*(*x*_2_) = *f*(*z*_1_) or *f*(*x*_2_) = *f*(*z*_2_). Without loss of generality, we can suppose *f*(*x*_2_) = *f*(*z*_1_). Since *z*_1_ is adjacent to every vertex of {*z*_2_, *y*_1_, *y*_2_} and {*x*_1_, *x*_2_} ∈ *E*(*X*[*Y*]), *f*(*z*_1_) is adjacent to every vertex of {*f*(*z*_2_), *f*(*y*_1_), *f*(*y*_2_), *f*(*x*_1_)}. Note that there is no vertex in *X*[*Y*] adjacent to 4 vertices. This is a contradiction. Hence *f* ∈ Aut(*X*[*Y*]) and so *f* is completely regular. If [*x*_1_]_*ρ*_*f*__ ≠ {*x*_1_}, then *f*(*x*_1_) = *f*(*z*_1_) or *f*(*x*_1_) = *f*(*z*_2_). Without loss of generality, we can suppose *f*(*x*_1_) = *f*(*z*_1_). Then *f*(*x*_2_) = *f*(*z*_2_). Otherwise, a similar argument as above will show that *f*(*x*_1_) is adjacent to every vertex of {*f*(*x*_2_), *f*(*y*_1_), *f*(*y*_2_), *f*(*z*_2_)}, which yield a contradiction. Thus *I*_*f*_≅*K*_4_. Since any endomorphism *f* maps a clique to a clique of the same size, *f*(*I*_*f*_) = *I*_*f*_. By Lemma 2, *f* is completely regular. Hence *P*_2_[*K*_2_] is End-completely-regular.

*Case 2.* *X* = *K*_1_ ∪ *K*_2_ and *Y* = *K*_2_. Let *f* ∈ End(*X*[*Y*]). If [*c*_1_]_*ρ*_*f*__ = {*c*_1_}, then [*c*_2_]_*ρ*_*f*__ = {*c*_2_}. Otherwise, *f*(*c*_2_)∈{*f*(*a*_1_), *f*(*a*_2_), *f*(*b*_1_), *f*(*b*_2_)}. Without loss of generality, we can suppose *f*(*c*_2_) = *f*(*a*_1_). Since any endomorphism *f* maps a clique to a clique of the same size and there is only one clique of size 4 in *X*[*Y*], {*f*(*a*_1_), *f*(*a*_2_), *f*(*b*_1_), *f*(*b*_2_)} = {*a*_1_, *a*_2_, *b*_1_, *b*_2_}. Note that {*c*_1_, *c*_2_} ∈ *E*(*X*[*Y*]). Then {*f*(*c*_1_), *f*(*c*_2_)} = {*f*(*c*_1_), *f*(*a*_1_)}∈ *E*(*X*[*Y*]). Thus *f*(*c*_1_)∈{*f*(*a*_1_), *f*(*a*_2_), *f*(*b*_1_), *f*(*b*_2_)}, which is a contradiction. Clearly, [*x*]_*ρ*_*f*__ = {*x*} for any *x* ∈ {*a*_1_, *a*_2_, *b*_1_, *b*_2_}. Hence *f* ∈ Aut(*X*[*Y*]) and so *f* is completely regular. If [*c*_1_]_*ρ*_*f*__ ≠ {*c*_1_}, then *f*(*c*_1_) = *f*(*t*) for some *t* ∈ {*a*_1_, *a*_2_, *b*_1_, *b*_2_}. Without loss of generality, we can suppose *f*(*c*_1_) = *f*(*a*_1_). Then *f*(*c*_2_)∈{*f*(*a*_2_), *f*(*b*_1_), *f*(*b*_2_)}. Thus *I*_*f*_≅*K*_4_. Hence *f*(*I*_*f*_) = *I*_*f*_ and *f* is completely regular. Consequently, (*K*_1_ ∪ *K*_2_)[*K*_2_] is End-completely-regular.

*Case 3.* *X* = 2*K*_2_ and *Y* = *K*_2_. Let *f* ∈ End(*X*[*Y*]). If [*x*]_*ρ*_*f*__ = {*x*} for any *x* ∈ *V*(*X*[*Y*]), then *f* ∈ Aut(*X*[*Y*]) and so *f* is completely regular. If *f*(*x*) = *f*(*y*) for some *x*, *y* ∈ *V*(*X*[*Y*]) with *x* ≠ *y*, without loss of generality, we can suppose *f*(*a*_1_) = *f*(*c*_1_). Since *b*_1_, *b*_2_, *c*_1_, *c*_2_ is a clique of size 4 in *X*[*Y*], *f*(*b*_1_), *f*(*b*_2_), *f*(*c*_1_), *f*(*c*_2_) is also a clique of size 4 in *X*[*Y*]. Note that *a*_2_, *d*_1_, *d*_2_ are adjacent to *a*_1_. Then *f*(*a*_2_), *f*(*d*_1_), *f*(*d*_2_) are adjacent to *f*(*a*_1_) = *f*(*c*_1_). Thus *f*(*a*_2_), *f*(*d*_1_), *f*(*d*_2_)∈{*f*(*b*_1_), *f*(*b*_2_), *f*(*c*_2_)} and *I*_*f*_≅*K*_4_. Hence *f*(*I*_*f*_) = *I*_*f*_ and *f* is completely regular. Consequently, (2*K*_2_)[*K*_2_] is End-completely-regular.

*Necessity*. We only need to show that *X*[*Y*] is not End-completely-regular in the following cases.

*Case 1*  (*X* = *K*_2_). Then *X*[*Y*] = *Y* + *Y*. By Lemma 4, *K*_2_[*Y*] is not End-completely-regular for the corresponding *Y*.

*Case 2*  (*X* = *P*_2_). Then *K*_2_ is a retract of *X*. Since *K*_2_[*Y*] is not End-completely-regular for *Y* = *P*_2_, 2*K*_1_, 2*K*_2_, *K*_1_ ∪ *K*_2_, by Lemma 12, *P*_2_[*Y*] is not End-completely-regular for the corresponding *Y*.

*Case 3*  (*X* = 2*K*_1_). Then *X*[*Y*] = 2*Y*. If *Y* is bipartite, then *X*[*Y*] is also bipartite. By Lemma 3, (2*K*_1_)[*Y*] is not End-completely-regular for the corresponding *Y*.

*Case 4*  (*X* = 2*K*_2_). Then *X*[*Y*] = 2(*Y* + *Y*). Since *Y* + *Y* is not End-completely-regular for *Y* = *P*_2_, 2*K*_1_, 2*K*_2_, *K*_1_ ∪ *K*_2_, by Lemma 13, (2*K*_2_)[*Y*] is not End-completely-regular for the corresponding *Y*.

*Case 5*  (*X* = *K*_1_ ∪ *K*_2_). Then *X*[*Y*] = *Y* ∪ (*Y* + *Y*). Since *Y* + *Y* is not End-completely-regular for *Y* = *P*_2_, 2*K*_1_, 2*K*_2_, *K*_1_ ∪ *K*_2_, by Lemma 13, (*K*_1_ ∪ *K*_2_)[*Y*] is not End-completely-regular for the corresponding *Y*.

Next we start to seek the conditions for a lexicographic product of bipartite graphs *X* and *Y* under which *X*[*Y*] is End-inverse.

**Theorem 15**

Let *X* and *Y* be two graphs. If *X*[*Y*] is End-inverse, then both *X* and *Y* are End-inverse.

**Proof**

Since *X*[*Y*] is End-inverse, *X*[*Y*] is End-regular. By Lemma 1, both *X* and *Y* are End-regular. To show that *X* is End-inverse, we only need to prove that the idempotents of End(*X*) commute.

Let *f*_1_ and *f*_2_ be two idempotents in End(*X*). Define two mappings *g*_1_ and *g*_2_ from *V*(*X*[*Y*]) to itself by

(5) $$\begin{array}{c} g_{1}\left(\left(x,y\right)\right)=\left(f_{1}\left(x\right),y\right)\;\forall \left(x,y\right)\in V\left(X\left[Y\right]\right),\\ g_{2}\left(\left(x,y\right)\right)=\left(f_{2}\left(x\right),y\right)\;\forall \left(x,y\right)\in V\left(X\left[Y\right]\right). \end{array}$$

Then *g*_1_ and *g*_2_ are two idempotents of End(*X*[*Y*]) and so *g*_1_ *g*_2_ = *g*_2_ *g*_1_, since *X*[*Y*] is End-inverse. For any (*x*, *y*) ∈ *V*(*X*[*Y*]), we have

(6) (g1g2)((x,y))=g1((f2(x),y))=((f1f2)(x),y)=(g2g1)((x,y))=((f2f1)(x),y).

Clearly, *f*_1_ *f*_2_ = *f*_2_ *f*_1_. Hence *X* is End-inverse.

Similarly, let *f*_3_ and *f*_4_ be two idempotents in End(*Y*). Define two mappings *g*_3_ and *g*_4_ from *V*(*X*[*Y*]) to itself by

(7) $$\begin{array}{c} g_{3}\left(\left(x,y\right)\right)=\left(x,f_{3}\left(y\right)\right)\;\forall \left(x,y\right)\in V\left(X\left[Y\right]\right),\\ g_{4}\left(\left(x,y\right)\right)=\left(x,f_{4}\left(y\right)\right)\;\forall \left(x,y\right)\in V\left(X\left[Y\right]\right). \end{array}$$

Then *g*_3_ and *g*_4_ are two idempotents of End(*X*[*Y*]) and so *g*_3_ *g*_4_ = *g*_4_ *g*_3_, since *X*[*Y*] is End-inverse. For any (*x*, *y*) ∈ *V*(*X*[*Y*]), we have

(8) (g3g4)((x,y))=(x,(f3f4)(y))=(g4g3)((x,y))=(x,(f4f3)(y)).

Clearly, *f*_3_ *f*_4_ = *f*_4_ *f*_3_. Hence *Y* is End-inverse, as required.

The next theorem characterizes the End-inverse lexicographic products of bipartite graphs.

**Theorem 16**

Let *X* and *Y* be two bipartite graphs. Then *X*[*Y*] is End-inverse if and only if *X*[*Y*] is one of *K*_1_[*K*_1_], *K*_1_[*K*_2_], *K*_2_[*K*_1_], and *K*_2_[*K*_2_].

**Proof**

*Necessity.* This follows directly from Lemma 6 and Theorem 15.

*Sufficiency*. It is easy to see that *K*_1_[*K*_1_] = *K*_1_, *K*_1_[*K*_2_] = *K*_2_[*K*_1_] = *K*_2_, and *K*_2_[*K*_2_] = *K*_4_ are End-inverse, since they are unretractive.

## References

[1] Wilkeit E (1996). Graphs with a regular endomorphism monoid. *Archiv der Mathematik*.

[2] Fan S (1996). On end-regular graphs. *Discrete Mathematics*.

[3] Hou H, Luo Y (2008). Graphs whose endomorphism monoids are regular. *Discrete Mathematics*.

[4] Hou H, Gu R, Li X End-completely-regular and end-inverse joins of graphs.

[5] Hou H, Luo Y, Fan X (2012). End-regular and end-orthodox joins of split graphs. *Ars Combinatoria*.

[6] Hou HL, Luo YF, Gu R (2010). The join of split graphs whose half-strong endomorphisms form a monoid. *Acta Mathematica Sinica*.

[7] Li W, Chen J (2001). Endomorphism—regularity of split graphs. *European Journal of Combinatorics*.

[8] Li W (2003). Graphs with regular monoids. *Discrete Mathematics*.

[9] Böttcher M, Knauer U (1992). Endomorphism spectra of graphs. *Discrete Mathematics*.

[10] Howie JM (1995). *Fundamentals of Semigroup Theory*.

[11] Kelarev AV, Ryan J, Yearwood J (2009). Cayley graphs as classifiers for data mining: the influence of asymmetries. *Discrete Mathematics*.

[12] Kelarev AV (2003). *Graph Algebras and Automata*.

[13] Knauer U (2011). *Algebraic Graph Theory: Morphisms, Monoids and Matrices*.

[14] Petrich P, Reilly NR (1999). *Completely Regular Semigroups*.

[15] Fan S (1997). End-regular graphs. *Journal of Jinan University*.

[16] Li W (2006). Split graphs with completely regular endomorphism monoids. *Journal of Mathematical Research and Exposition*.

[17] Fan SH (1995). The endomorphism monoid of the lexicographic product of two graphs. *Acta Mathematica Sinica*.

